# Neuroaxonal Degeneration as a Converging Mechanism in Motor Neuron Diseases (MNDs): Molecular Insights into RNA Dysregulation and Emerging Therapeutic Targets

**DOI:** 10.3390/ijms26157644

**Published:** 2025-08-07

**Authors:** Minoo Sharbafshaaer, Roberta Pepe, Rosaria Notariale, Fabrizio Canale, Alessandro Tessitore, Gioacchino Tedeschi, Francesca Trojsi

**Affiliations:** 1 First Division of Neurology, Department of Advanced Medical and Surgical Sciences, University of Campania “Luigi Vanvitelli”, 80138 Naples, Italy; 2 Department of Advanced Medical and Surgical Sciences, MRI Research Center, University of Campania “Luigi Vanvitelli”, 81038 Naples, Italy

**Keywords:** motor neuron diseases, RNA-binding proteins, protein aggregation, mitochondrial dysfunction, DNA repair, kinase signaling, axonal transport, neuroinflammation, neurofilament biomarkers, targeted therapies

## Abstract

Motor Neuron Diseases (MNDs) such as Amyotrophic Lateral Sclerosis (ALS), Primary Lateral Sclerosis (PLS), Hereditary Spastic Paraplegia (HSP), Spinal Muscular Atrophy with Respiratory Distress Type 1 (SMARD1), Multisystem Proteinopathy (MSP), Spinal and Bulbar Muscular Atrophy (SBMA), and ALS associated to Frontotemporal Dementia (ALS-FTD), have traditionally been studied as distinct entities, each one with unique genetic and clinical characteristics. However, emerging research reveals that these seemingly disparate conditions converge on shared molecular mechanisms that drive progressive neuroaxonal degeneration. This narrative review addresses a critical gap in the field by synthesizing the most recent findings into a comprehensive, cross-disease mechanisms framework. By integrating insights into RNA dysregulation, protein misfolding, mitochondrial dysfunction, DNA damage, kinase signaling, axonal transport failure, and immune activation, we highlight how these converging pathways create a common pathogenic landscape across MNDs. Importantly, this perspective not only reframes MNDs as interconnected neurodegenerative models but also identifies shared therapeutic targets and emerging strategies, including antisense oligonucleotides, autophagy modulators, kinase inhibitors, and immunotherapies that transcend individual disease boundaries. The diagnostic and prognostic potential of Neurofilament Light Chain (NfL) biomarkers is also emphasized. By shifting focus from gene-specific to mechanism-based approaches, this paper offers a much-needed roadmap for advancing both research and clinical management in MNDs, paving the way for cross-disease therapeutic innovations.

## 1. Introduction

Motor neuron diseases (MNDs) are a heterogeneous group of neurodegenerative disorders characterized by progressive loss of motor neurons in the brain, brainstem, and spinal cord. While Amyotrophic Lateral Sclerosis (ALS) represents the most common form, other MNDs, including Primary Lateral Sclerosis (PLS), Hereditary Spastic Paraplegia (HSP), Spinal Muscular Atrophy with Respiratory Distress Type 1 (SMARD1), Multisystem Proteinopathy (MSP), Spinal and Bulbar Muscular Atrophy (SBMA), and ALS associated to Frontotemporal Dementia (ALS-FTD), contribute significantly to the clinical and biological complexity of these diseases spectrum [1,2].

Despite differences in genetic causes, clinical manifestations, and age of onset, these disorders converge pathologically on a common endpoint: progressive neuroaxonal degeneration. Axonal degeneration often precedes motor neuron soma death and is a critical determinant of functional decline and disability [3]. Understanding the molecular events that trigger axonal degeneration across MNDs offers valuable insights into shared disease mechanisms and potential therapeutic targets (Table 1).

Recent advances have identified several key molecular pathways involved in the pathogenesis of axonal degeneration. These include RNA dysregulation, resulting from mutations in RNA-binding proteins, such as TDP-43 (TARDBP), FUS, and components of the RNA exosome (EXOSC3) [4,5]; protein misfolding and aggregation, involving proteins such as Valosin-Containing Protein (VCP), androgen receptor (AR), and SOD1 [6,7]; defects in axonal transport, linked to mutations in *DYNC1H1*, *BICD2*, and *KIF5A* [8]. Biomarkers, such as neurofilament light chain (NfL), have emerged as encouraging tools for dysfunction, often secondary to impaired oxidative phosphorylation and calcium buffering, notably in SPG7- and C9orf72-associated diseases [9]; and neuroinflammatory mechanisms, including glial cell activation and T cell-mediated damage [10] (Table 2).

In addition to these molecular triggers, the use of biomarkers, such as NfL, has emerged as a promising tool for tracking axonal damage and disease progression across multiple forms of MNDs [11]. Furthermore, emerging therapies, including antisense oligonucleotides (ASOs), autophagy enhancers, and mitochondrial stabilizers, are increasingly being designed to target these common molecular pathways rather than focusing solely on the specific genetic mutations.

**Figure 1:**
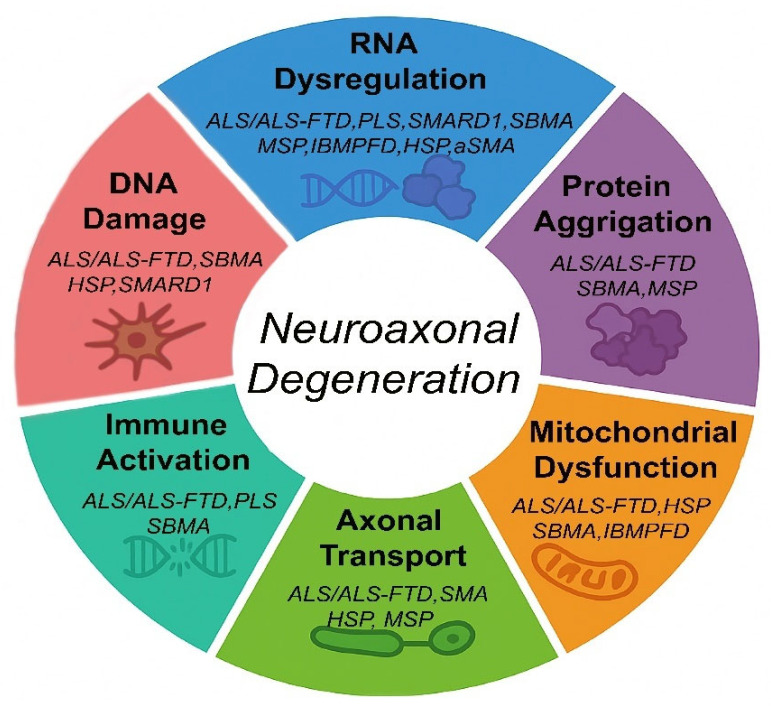
Common molecular mechanisms driving neuroaxonal degeneration within MNDs. Note: Key pathogenic pathways, including RNA dysregulation, protein aggregation, mitochondrial dysfunction, axonal transport defects, DNA damage, and immune activation, converge on neuroaxonal degeneration across ALS/ALS-FTD, PLS, SMA, SBMA, HSP, PCH1, MSP, IBMPFD, and SMARD1.

This review aims to explore the molecular mechanisms underlying axonal degeneration in MNDs. It identifies common molecular targets by combining new research, including information from iPSC-derived models on convergent pathogenic pathways (like RNA dysregulation, proteostasis failure, mitochondrial dysfunction, kinase signaling imbalances, DNA damage, axonal transport deficits, and immune activation). It places a strong emphasis on cross-disease treatment strategies designed to preserve neuroaxonal integrity (Figure 1).

## 2. Molecular Mechanisms of Neuroaxonal Degeneration in MNDs

### 2.1. RNA Dysregulation

RNA dysregulation is a fundamental pathological feature of MNDs, disrupting mRNA splicing, transport, stability, and localized translation, processes vital for maintaining axonal integrity and synaptic function in motor neurons. In ALS, mutations in genes like *TARDBP*, *FUS*, and *C9orf72* cause mislocalization of RNA-binding proteins (RBPs), formation of toxic RNA aggregates, and impaired nucleocytoplasmic transport, ultimately contributing to protein aggregation, stress granule formation, and axonal degeneration [4,12,13]. Similar disturbances occur in other MNDs: mutations in hnRNPA1 and hnRNPA2B1 promote aggregation-prone ribonucleoprotein granules [13]; IGHMBP2 deficiency in SMA disrupts ribosome biogenesis and mRNA processing [14]. Concurrently, RNA dysregulation is not a secondary effect but a primary driver of motor neuron vulnerability, highlighting the therapeutic potential of targeting RNA metabolism using approaches such as antisense oligonucleotides and RBP modulators.

A major cause of ALS is RNA dysregulation, which primarily involves mislocalized RNA-binding proteins like TDP-43 and FUS that aggregate in the cytoplasm and interfere with translation, splicing, and transport [12]. Accordingly, dipeptide repeats and toxic RNA foci are produced by exanucleotide repeat expansions in C9orf72, which hold back RNA granule dynamics and nucleocytoplasmic transport [15,16]. iPSC models and single-cell analyses reveal widespread splicing defects, mRNA mislocalization, and disrupted mitochondrial transport and bioenergetics [17]. These findings highlight RNA processing defects as initiating factors in motor neuron degeneration, supporting RNA-targeted therapies, including ASOs and RBP modulators, as hopeful treatments [18].

RNA dysregulation may be a factor in PLS, like in ALS. Though mislocalized TDP-43 and altered gene expression linked to splicing, axonal growth, and immune signaling in the motor cortex are increasingly evident [19,20]. Splicing factor alterations and transcripts linked to stress granules are revealed by high-throughput sequencing, which points to a persistent disturbance of RNA homeostasis [20,21]. Although there are no RBP mutations in PLS, these results suggest that RNA misprocessing plays a role in the degeneration of upper motor neurons, which may pave the way for RNA-targeted treatments.

Recent research indicates that RNA dysregulation, particularly in SPG11 and SPG7 subtypes, plays a role in the pathophysiology of HSP. When SPG11 is lost, lysosomal function and axonal maintenance are impacted, splicing is disrupted, spliceosomal gene expression is changed, and transcripts linked to synaptic functions are impaired [22,23]. Stress granule-associated RNAs and RNA-binding proteins are misexpressed in SPG11-mutant models, which connect RNA instability to abnormalities in proteostasis [24]. Although RNA is not directly controlled by SPG7, mitochondrial stress decreases RNA stability and translation in neurons that lack SPG7 [25]. These results point to new therapeutic targets by establishing RNA dysregulation as a major cause of axonal degeneration in complex HSP.

SMA refers to a group of disorders affecting lower motor neurons. Among these disorders, SMARD1 is a spinal muscular atrophy with its clinical entity. It is also referred to as DSMA1 (distal spinal muscular atrophy type 1) and results from biallelic mutations in *IGHMBP2*, a helicase essential for transcription termination and ribosome biogenesis. Its loss disrupts RNA processing, causing incomplete transcript maturation, cryptic intron accumulation, and aberrant splicing of motor neuron-specific genes [26]. Widespread RNA metabolism abnormalities are seen in iPSC-derived motor neurons, specifically affecting transcripts essential for mitochondrial and neuromuscular junction function [27]. Axonal vulnerability, delayed translation, and proteostasis failure are all consequences of impaired helicase activity, which also impairs ribosomal RNA processing [28,29]. According to these results, RNA dysregulation plays a key role in SMARD1 pathogenesis.

MSP is a rare, genetically diverse disorder associated with mutations in VCP, HNRNPA1, and HNRNPA2B1 that causes motor neuron degeneration, muscle weakness, and FTD. These genes produce cofactors or RNA-binding proteins that are essential for transport, stress granule dynamics, and RNA splicing. Liquid–liquid phase separation (LLPS) is impaired by mutations in hnRNPA1 and hnRNPA2B1, which promote the development of persistent cytoplasmic RNA-protein aggregates with prion-like characteristics that are made worse by disease mutations [30,31]. Multisystem proteinopathy (VCP-MSP), an autosomal dominant adult-onset disorder affecting multiple tissues, is caused by VCP mutations that interfere with important cellular functions such as proteostasis, stress granule clearance, and genome integrity [32]. In recent studies, genes controlling proteostasis, mitochondrial activity, and the cytoskeleton have been shown to exhibit altered expression [21,33]. A major pathogenic factor in MSP is RNA dysregulation, which supports RNA-targeted treatments such as antisense tactics and phase separation modulators.

Kennedy’s disease, or SBMA, is brought on by an expansion of the CAG repeat in the AR gene, which results in a polyglutamine expanded AR protein that interferes with RNA metabolism and other cellular functions. Axonal mRNA trafficking, RNA export, and pre-mRNA splicing are all disrupted by mutant AR’s abnormal interactions with RNA-binding proteins, including hnRNPA1, SFPQ, and TDP-43, which capture them into nuclear and cytoplasmic inclusions [34,35]. Widespread RNA homeostasis disruption is revealed by transcriptomic profiling of motor neurons derived from SBMA patients. This disruption includes changes in the expression of splicing regulators, non-coding RNAs, and stress granule components. Along with mitochondrial dysfunction and the breakdown of proteostasis, these RNA abnormalities also lead to motor neuron degeneration [36]. Androgen deprivation, gene silencing techniques, and RNA-modulating treatments like ASOs and splicing-corrective interventions are examples of emerging therapeutic approaches. The therapeutic landscape for SBMA and related neuromuscular diseases is set to rapidly evolve as a result of these strategies’ advancement into clinical application [37].

IBMPFD is a multisystem neurodegenerative disorder commonly caused by *VCP* mutations. VCP is essential for RNA quality control, including stress granule disassembly and degradation of aberrant ribonucleoprotein complexes. Mutant VCP impairs granule clearance, leading to toxic cytoplasmic aggregates enriched with RNA-binding proteins like TDP-43, FUS, and hnRNPA1, which disrupt RNA splicing, export, and translation [38,39]. Transcriptomic analyses of IBMPFD patient-derived cells and muscle biopsies reveal altered RNA surveillance and processing factors expression, highlighting widespread RNA homeostasis disruption [40]. RNA dysregulation is linked to both motor neuron dysfunction and muscle degeneration. These deficiencies worsen under cellular stress, where mutant VCP fails to resolve granules and promote recovery. According to recent research, VCP controls cellular functions differently depending on the context and type of cell, indicating that RNA dysregulation plays a significant role in the pathophysiology of IBMPFD and can be a target for treatment [41].

RNA dysregulation seen in Spinal Muscular Atrophy Lower Extremity Dominant (SMALED), SMA adult-onset associated with BICD2 and DYNC1H1 mutations, is caused by impaired axonal RNA transport. BICD2 and DYNC1H1 are essential for mRNA trafficking along microtubules in motor neurons, despite not being classical RNA-binding proteins. A mechanism of progressive motor neuron degeneration caused by defective RNA localization is supported by their dysfunction, which interferes with local translation at neuromuscular junctions [42]. According to recent research on motor neurons derived from iPSCs, these transport deficits cause changes in the spatial transcriptome, including the mislocalization of transcripts required for cytoskeletal stability, synaptic plasticity, and mitochondrial maintenance, all are essential to meet the high energy demands of axons. Remarkably, several transcripts connected to hereditary axonopathies were discovered among the mRNA species that were elevated in motor axons [43]. Furthermore, compensatory upregulation of splicing regulators and stress granule activation have been observed in BICD2- and DYNC1H1-mutant models, indicating that RNA dysregulation contributes to motor neuron degeneration in adult-onset genetic SMA [43,44].

#### 2.1.1. RNA Metabolism

Transcription, splicing, editing, nuclear export, localization, translation, and degradation are all components of the complex process of RNA metabolism. It is becoming more widely acknowledged that disruption of these delicately regulated processes causes extensive transcriptomic instability and is a key factor in motor neuron degeneration in a variety of MNDs. Furthermore, mutations in TDP-43, FUS, and C9orf72 affect splicing and RNA surveillance, which disrupts RNA metabolism in ALS and ALS-FTD. TDP-43 and FUS are normally nuclear, but they mislocalize to the cytoplasm and form harmful aggregates, making motor neurons vulnerable and leading to widespread RNA processing defects [45]. These mutations change RNA-binding affinity and nucleocytoplasmic shuttling, which leads to the formation of toxic RNA foci and stress granules [1,12,13]. Transcription termination and ribosome biogenesis are impacted by IGHMBP2 loss in SMARD1, compromising the stability and maturation of mRNAs essential for neuromuscular junction function and mitochondrial integrity [26,27,28]. Similarly, mutations in EXOSC3 in pontocerebellar hypoplasia affect the RNA exosome complex, disrupting RNA processing and degradation [21].

Whereas hnRNPA1/2B1 mutations push the aggregation of defective RNA granules through altered liquid–liquid phase separation, mutations in VCP prevent RNA turnover in MSP and IBMPFD by inhibiting the disintegration of aberrant ribonucleoprotein (RNP) complexes and stress granules [30,32,46]. Together, these deficiencies raise neurotoxicity and disrupt RNA quality control, particularly in long-projecting, metabolically active motor neurons.

RNA metabolism dysfunction plays a key part in the pathophysiology of MNDs. In motor neurons with high metabolic demands, disruption of especially adjusted RNA processing steps results in axonal degeneration, toxic aggregate formation, and reduced transcript stability. For genetically varied types of MND, RNA metabolic pathway targeting could be a therapeutic intervention option.

#### 2.1.2. RBP Pathology

**Figure 2:**
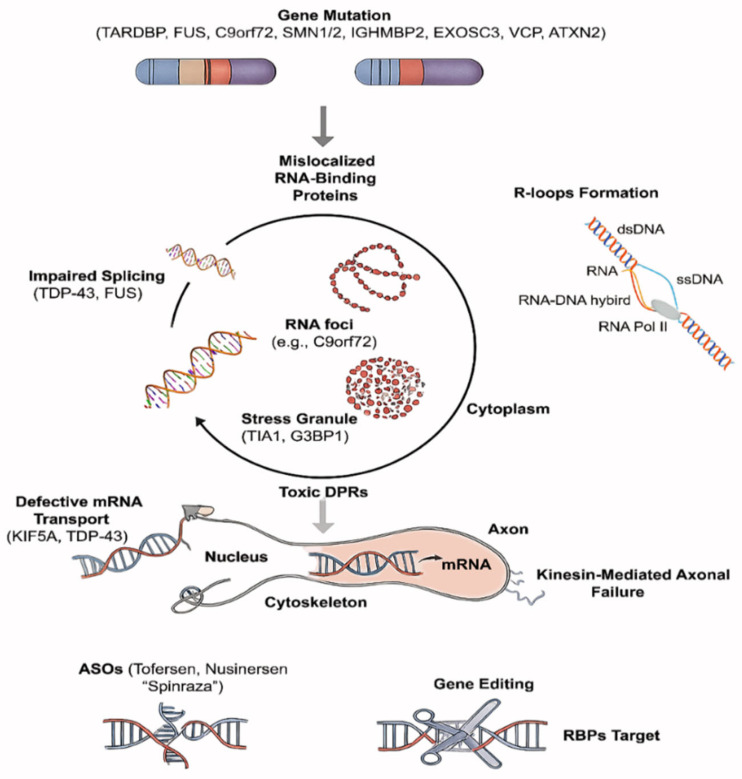
RNA dysregulation in motor neuron diseases: mechanisms and therapeutic targets. Note: Mutations in RNA-binding proteins (TARDBP, FUS, C9orf72, SMN1/2, IGHMBP2, EXOSC3, VCP, ATXN2) lead to their mislocalization, resulting in impaired splicing, RNA foci, stress granules, and R-loop formation. These disruptions impair mRNA transport and translation, contribute to toxic dipeptide repeat protein production, and culminate in axonal dysfunction. Therapeutic approaches include ASOs and gene-editing strategies targeting RNA metabolism.

A major factor in MNDs is pathological changes in RNA-binding proteins (RBPs), such as mislocalization, aggregation, and abnormal post-translational modifications. The hallmark cytoplasmic inclusions of TDP-43 and FUS in ALS are triggered by abnormal phosphorylation, acetylation, and ubiquitination as well as disrupted nuclear import, which impairs neuronal function [14,45]. In MSP and ALS-FTD, mutant forms of hnRNPA1 and hnRNPA2B1 accumulate in cytoplasmic stress granules, where they stop RNA processing and sequester essential RBPs. These mutant forms also go through pathological phase transitions [30]. These RBPs frequently have prion-like domains that help them aggregate and possibly spread between cells. Furthermore, RBP dysfunction impairs chromatin organization and RNA-DNA hybrid resolution (R-loops), which both lead to DNA damage [47] (Figure 2). Abnormal interactions between the mutant androgen receptor and TDP-43, SFPQ, and other RBPs in SBMA cause them to be sequestered into toxic inclusions that impair their physiological functions in RNA metabolism [34,35,36]. These RBP aggregates enhance cytoskeletal instability and stress granule persistence, besides affecting local translation and axonal maintenance.

A claimed treatment approach for MNDs is to target the pathological behavior of RBPs, such as stopping aggregation, re-establishing nucleocytoplasmic transport, and modifying RNA interactions. Increasing autophagy has the potential to improve neuronal function, restore homeostasis, and decrease cytoplasmic RBP accumulation [48], highlighting RBP dysregulation as a crucial therapeutic target for a variety of motor neuron disorders.

#### 2.1.3. Axonal Transport

In motor neurons, long-distance transport of mRNAs, proteins, organelles, and signaling molecules depends on axonal transport. Microtubule-based motors, such as dyneins and kinesins, mediate this process by bidirectionally transporting axonal cargo trafficking within the nervous system in cells [49,50]. The mislocalization of essential cargoes, mitochondria, mRNAs, and neurotrophic signals, occurs in MNDs when the axonal transport is disrupted by mutations in DYNC1H1, BICD2, KIF5A, and TUBA4A [51].

Axonal transport deficits in ALS cause degeneration by preventing neuroprotective cargoes from reaching distal axons and synapses. TDP-43 aggregates block transport granules and mitochondrial trafficking, while C9orf72 expansions alter microtubule dynamics and dynein function [14,15,49]. Likewise, impaired retrograde transport results in distal axonal energy deficits and stress granule accumulation in adult-onset SMA associated with DYNC1H1 or BICD2, which causes neuromuscular junction instability [43].

Furthermore, dynein-mediated transport of autophagic vesicles is impaired by mutant VCP in MSP and IBMPFD, which leads to the accumulation of damaged organelles and protein aggregates [51]. IGHMBP2 deficiency in SMARD1 impairs localized translation and mitochondrial protein synthesis by impacting the axonal distribution of transcripts and ribosomes [52].

In particular, the final common pathway in MNDs is axonal transport failure. Currently, studies are being conducted on therapeutic strategies that aim to enhance motor protein function, restore microtubule dynamics, and improve organelle trafficking.

### 2.2. Prion-like Propagation of Misfolded Proteins in MNDs

Recent evidence has revealed that MNDs involve the prion-like propagation of misfolded proteins, mechanisms once thought exclusive to transmissible prion diseases [53]. In MNDs, prion-like proteins spread throughout the central nervous system, spreading disease along anatomically connected pathways, in contrast to classical prions (e.g., PrP^Sc^), which transmit between individuals [53,54]. TDP-43, FUS, SOD1, hnRNPA1, hnRNPA2B1, VCP, and polyglutamine-expanded AR are among the disease-associated proteins that experience abnormal phase transitions and form aggregation-prone and β-sheet-rich conformers that cause cytoplasmic inclusions, template misfolding, and disturb cellular homeostasis [55]. These misfolded proteins build up in motor neurons and glia in ALS, SBMA, MSP, and IBMPFD, which interfere with nucleocytoplasmic transport, RNA metabolism, autophagy, and the removal of stress granules [56]. Research utilizing iPSC models, transgenic animals, and patient-derived tissues demonstrates how they spread through axonal transport, synaptic transmission, or exosomal release [57]. Early aggregate formation, seeding, or intercellular transmission as viable therapeutic targets by highlighting prion-like propagation as a convergent pathogenic mechanism across various MNDs [58,59] (Table 3).

In ALS, increasing evidence indicates that key disease-related proteins, such as TDP-43, SOD1, and FUS, undergo prion-like misfolding and intercellular propagation, playing a central role in disease progression. Misfolded TDP-43, found in over 95% of ALS cases, undergoes liquid–solid phase transition, forming insoluble cytoplasmic aggregates that convert native TDP-43 into pathological forms via a mechanism known as templated seeding [12,60]. Similarly, mutant forms of SOD1 and FUS assemble into amyloid-like fibrils that propagate through motor neuron circuits by exosomes, tunneling nanotubes, or synaptic connections, thereby promoting non-cell-autonomous toxicity [58]. These protein aggregates impair RNA metabolism, nucleocytoplasmic transport, and proteostasis, particularly under oxidative stress or when autophagic pathways are compromised [61]. Notably, recent iPSC-derived motor neurons in postmortem human tissue and transgenic models confirm that these aggregates retain seeding capacity and induce pathology in recipient neurons. ASOs and small molecules that target RNA G-quadruplexes can be used to rescue models with nuclear import defects and C9orf72 hexanucleotide repeat expansions caused by repeat RNA structures [62]. Improving aggregate clearance, stopping intercellular spread, or focusing on early aggregation events. These tactics, especially those that use ASOs, are becoming more and more popular as practical ways to slow or stop the progression of ALS [63].

In PLS, evidence suggests that prion-like propagation of misfolded proteins, particularly TDP-43, may contribute to disease pathogenesis. Cortical TDP-43 mislocalization and phosphorylated inclusions have been found in postmortem investigations, suggesting an overlap with ALS pathology [64]. Stress granule-associated transcripts and splicing regulators, which are known to undergo liquid–liquid phase separation and can form self-propagating aggregates under chronic cell stress [65]. These results lend support to the idea that slow, compartmentalized RBP aggregation and seeding may be the cause of cortical neurodegeneration, even though the degree of prion-like spread in PLS is less clear than in ALS [66]. This changing viewpoint places PLS in line with other neurodegenerative proteinopathies and emphasizes how future treatments may be able to target protein propagation.

Despite the genetic and clinical diversity of HSP, particularly in the SPG11 and SPG7 subtypes, disease mechanisms are influenced by prion-like propagation and protein misfolding. Loss of function in SPG11, which codes for the lysosomal protein spatacsin, results in misregulated RNA-binding proteins such as hnRNPA1, autophagic impairment, and stress granule accumulation. In membraneless compartments created under stress, these modifications promote the aggregation and self-seeding of RBPs [67]. These persistent cytoplasmic aggregates can spread among interconnected neurons and are resistant to removal. Chronic mitochondrial stress has been found to worsen RNA granule aggregation and misfolded protein accumulation in SPG7, which codes for the mitochondrial protease paraplegin, thereby indirectly promoting aggregation-prone conditions [68]. In contrast to SPAST cells, SPG7 patient cells exhibit mitochondrial dysfunction, which provides additional support to this mechanism [69]. Although research on the extent of transmissible pathology in HSP is still ongoing, these results support more general models of phase transition-driven neurodegeneration and implicate prion-like dynamics as a contributing factor in complex HSP.

Although RNA metabolism and ribosome biogenesis defects are the main causes of SMARD1, new research indicates that prion-like protein aggregation also plays a role in the development of this MND [14,70]. Ribosome delay, transcriptional stress, and abnormal RNP complex accumulation, including RBPs with prion-like domains, such as hnRNPA2B1, FUS, and G3BP1, are caused by IGHMBP2 deficit [54]. When under stress or with proteasome impairment, these granules can change from dynamic liquid phases to solid fibrillar inclusions [28]. It has been suggested that protein disaggregases like Hsp104 can reverse these abnormal phase transitions [54]. Persistent aggression-prone RBPs and impaired clearance are similar to prion-like dynamics in ALS and MSP, indicating shared vulnerabilities in motor neurons, even though intercellular spread is not fully established in SMARD1 [53,71]. These results highlight the interaction of proteostasis collapse, RNA dysregulation, and the therapeutic potential of SMARD1 protein aggregate targeting.

Mutations in VCP, HNRNPA1, and HNRNPA2B1 proteins, which are involved in RNA metabolism, stress granule dynamics, and proteostasis, are linked to MSP, which impacts skeletal muscle, bone, and motor neurons. Human motor neurons have mutations in their low-complexity, prion-like domains that exhibit self-seeding, aggression-prone conformations, and stress-specific RBP responses as a result of the promotion of liquid–solid phase separation [72,73]. Mutant hnRNPA1/2B1 and VCP pathological inclusions show β-sheet-rich fibrils, bring in native proteins, and spread intercellularly through axonal transport and exosomes [31,74]. Especially in long-projecting motor neurons, these aggregates interfere with RNA splicing, nucleocytoplasmic transport, and overburden autophagy and proteasomal pathways [73]. MSP-linked mutations enhance RBP misfolding and spread the toxic assemblies [73]. Positioning prion-like propagation as central with targeting aggregation kinetics or granule resolution mechanisms holds promise for MSP therapy.

Mutant AR causes Kennedy’s disease (SBMA) with a polyQ expansion that creates oligomeric and fibrillar aggregates in muscle cells and motor neurons, especially during development and prepuberty [75]. Co-regulators, transcription factors, and RBPs such as TDP-43 and hnRNPA1 are sequestered by these aggregates, upsetting transcriptional homeostasis, resulting in a self-replicating aggregation cascade [76]. In line with prion-like behavior, polyQ-AR aggregates recruit wild-type AR and spread misfolding through exosomes or direct contact, as demonstrated by experimental models and patient-derived cells. This leads to motor dysfunction, early death, muscle denervation, the upregulation of atrogenes and autophagy genes, the change from glycolytic to oxidative fiber type, the transition from IIb to IIa/IIx fiber type and mitochondrial dysfunction [75,77]. Protease and autophagy pathway impairments further improve aggregate stability and dissemination. According to these results, SBMA is a polyQ-driven proteinopathy with prion-like propagation dynamics, suggesting that it may be treated by focusing on the misfolding and aggregation of expanded polyQ proteins [76].

Multisystem degeneration and prion-like spread of misfolded proteins are hallmarks of IBMPFD, which is brought on by VCP mutations. Mutant VCP disrupts proteostasis and stress granule clearance, which causes RNA-binding proteins like TDP-43, hnRNPA1, and FUS to accumulate in the cytoplasm and exhibit prion-like, self-templating aggregation [78]. VCP-mutant models and patient-derived tissues have demonstrated that these aggregates can attract native proteins and spread intercellularly through exosomes or direct contact [79,80]. A toxic feedback loop is created when aggregate accumulation is made worse by compromised autophagy and proteasomal pathways [80,81]. These results support treatment approaches that focus on aggregate clearance and preventing intercellular spread, highlighting prion-like propagation as a major pathogenic mechanism in IBMPFD [59,80,81,82].

While adult-onset SMA, which is linked to BICD2 and DYNC1H1 mutations, is typically categorized as an axonal transport disorder, intracellular RNA granule trafficking may create an environment that facilitates the aggregation and spread of prion-like proteins. When BICD2 and DYNC1H1 are not functioning properly, stalled ribonucleoprotein complexes accumulate in the cytoplasm. These proteins are necessary for the microtubule-based transport of RBPs and stress granules [83]. The prion-like domains in these failed granules, which have been enhanced with RBPs like TDP-43, hnRNPA2B1, and G3BP1, are susceptible to pathological phase transitions. These transitions interrupt cellular homeostasis by changing from dynamic liquid phases to solid or gel-like states [84]. iPSC-derived neurons and mutant DYNC1H1 models exhibit persistent RNP granules and impaired clearance, similar to classical proteinopathies, despite the fact that intercellular propagation of aggregates in adult-onset SMA is not completely confirmed [85]. These results suggest that the pathogenic framework of this uncommon MND extends beyond axonopathy and includes secondary prion-like dynamics associated with impaired transport and granule resolution [86].

### 2.3. Kinase Signaling Abnormalities

Neuronal survival, synaptic function, axonal maintenance, and stress responses are all regulated by kinase-mediated signaling pathways. There is growing evidence that the pathophysiology of MNDs is associated with kinase dysregulation, including aberrant phosphorylation of RBPs, compromised stress cascades, and dysregulated MAPK, AKT, and ERK signaling [87,88]. Through changes in cytoskeletal dynamics, axon regeneration, apoptotic sensitivity, and neuroinflammation that have been frequently caused by genetic mutations in transport, proteostasis, or mitochondrial genes, these alterations could increase based on the disease progression [89]. Studies in ALS, SBMA, MSP, and HSP (SPG11, SPG7) reveal constitutive activation or mislocalization of kinases such as p38 MAPK, JNK, and GSK3β, which results in pathological phosphorylation of tau, TDP-43, and neurofilaments, impairing intracellular trafficking and stimulating neurotoxicity [90]. Additionally, mutations in *VCP*, *DYNC1H1*, and *IGHMBP2* impair upstream kinase regulation, linking RNA dysregulation and defective transport to signal transduction failure [91]. Knowledge of these alterations may improve treatments by targeting aberrant phosphorylation, restoring kinase balance, or modifying neurodegenerative signaling pathways. It also sheds light on convergent disease mechanisms.

In ALS motor neuron degeneration, dysregulated kinase signaling like irregular activation of p38 MAPK, ERK1/2, AKT, and GSK3β, interferes with RNA metabolism, protein aggregation, axonal integrity, and inflammatory responses [92]. Hyperphosphorylation of TDP-43, neurofilaments, and stress-responsive proteins promotes neuronal apoptosis and neuroinflammation, while dysregulated AKT and ERK signaling impairs axonal transport and synaptic function [92]. Mutant SOD1 and C9orf72 DPR proteins disrupt upstream kinase regulation, linking genetic pathology to abnormal phosphorylation [93]. Postmortem and iPSC-derived motor neuron studies confirm that kinase hyperactivation correlates with disease severity and vulnerability [92,94]. Inhibitors target p38 MAPK and GSK3β are currently being researched as a potential treatment strategy for ALS [95].

Progressive cortical pathology of PLS is linked to abnormalities in kinase signaling, it means increasing MAPK and JNK pathways as well as stress-responsive kinases linked to neuronal apoptosis and axonal degeneration that revealed by transcriptomic and proteomic analyses [96]. Although dysregulated phosphorylation of cytoskeletal proteins and altered GSK3β and ERK1/2 expression suggest contributions to TDP-43 dysregulation, splicing alterations, and corticospinal tract degeneration, even in the absence of classic inclusion pathology, elevated p38 MAPK activity in cortical pyramidal neurons correlates with neuroinflammation and glial activation [97]. kinase-targeted neuroprotective techniques may be considered as a possible PLS treatment strategy.

SPG11 and SPG7 dysfunction subtypes of HSP have been linked to abnormalities in kinase signaling and stress kinases like p38 MAPK, JNK, and GSK3β to become hyperactivated, which changes the phosphorylation of cytoskeletal and axonal maintenance proteins and dysregulates mTOR and AKT signaling; under metabolic stress, this modification reduces autophagosome clearance and neuronal survival [98]. Secondary kinase dysregulation is associated with SPG7-related mitochondrial dysfunction, and increased ERK1/2 and calcium-dependent kinase activity contribute to axonal swellings and neuronal degeneration [99]. Proteostasis collapse and RBP dysfunction may interact with these kinase disruptions, which worsen axonopathy. While targeting dysregulated kinase pathways could an effective method for influencing the disease severity.

IGHMBP2 deficiency disrupts transcription termination and ribosome biogenesis, indirectly activating stress-responsive kinase pathways such as p38 MAPK, JNK, and GSK3β in response to accumulated RNA-protein complexes, proteostasis overload, and various cellular stressors, including oxidative stress, endoplasmic reticulum stress, growth factors, cytokines, and hormones. Kinase signaling abnormalities increase neuronal stress and degeneration in SMARD1 due to abnormalities in kinase signaling [100]. Abnormal AKT and ERK1/2 activity lead to axonal retraction and mitochondrial dysfunction, while increased p38 MAPK activity is associated with apoptosis, neuroinflammation, and impaired neuromuscular junction maintenance in iPSC-derived motor neurons and transgenic models [100,101]. The dysregulation and degeneration of RNA are deepened by these kinase imbalances.

MSP includes abnormal kinase signaling plus to the growth of RNA-binding proteins and the collapse of proteostasis. Although stress-responsive kinases such as p38 MAPK, JNK, and ERK1/2 are activated when mutant VCP challenges with autophagic and proteasomal pathways, then raises inflammation and causes misfolded proteins made phosphorylated [101]. HNRNPA1 and HNRNPA2B1 mutations increase the hyperphosphorylation of prion-like domains, increasing phase separation and stress granule persistence. This creates feedback loops that disrupt neuronal signaling by dysregulating mRNA transcripts encoding kinases [102]. Dysregulation of adenine nucleotide translocase results in impaired ADP/ATP translocation across mitochondrial membranes, and pathogenic mutations and VCP knockdown induce mitochondrial uncoupling [103]. iPSC-derived neurons and VCP-mutant models declared Kinase dysregulation is emphasized as a significant cause of neuronal vulnerability, cytoskeletal instability, and mitochondrial specially highlighting its potential role for treatment.

The ERK1/2, AKT, and JNK pathways are abnormally impacted by mutant polyQ-expanded AR in SBMA, which alters phosphorylation dynamics and downstream gene expression [104]. Furthermore in motor neurons, hyperactivation of AKT and p38 MAPK leads to apoptosis, DNA damage, mitochondrial dysfunction, and instability of the neuromuscular junction [104]. Regardless of AR-mediated transcription, stress-responsive kinases phosphorylate mutant AR at particular residues, increasing its aggregation and transcriptional toxicity [105]. Dysregulated kinase pathways are highlighted as therapeutic targets in SBMA by patient-derived muscle biopsies, which demonstrate that kinase modulation increases muscle strength and slows the progression of the disease [105,106].

Kinase signaling dysregulation has been identified as a significant secondary contributor to neurodegeneration and muscle pathology in IBMPFD, which is caused by dominant VCP mutations. The pathological phosphorylation of RBPs, cytoskeletal proteins, and mitochondrial regulators is caused by p38 MAPK, JNK, and ERK1/2 being activated by persistent stress caused by mutant VCP’s impairment of autophagy and proteasomal clearance [107]. Developmental stage, cell type, and anatomical location all affect the consequences of MAPK/ERK activation. This abnormal signaling promotes TDP-43 mislocalization, stress granule persistence, and inflammatory responses, which in turn promote inclusion formation and neurotoxicity [87]. VCP dysfunction is linked to widespread signaling collapse by kinase imbalances in muscle and neuronal tissues, which further impair nuclear cytoplasmic transport and axonal stability. The p38 and ERK pathways are highlighted as therapeutic targets in recent patient-derived and animal studies to reduce proteostatic overload and degeneration [108]. Interestingly, the MAPK/ERK pathway also controls apoptosis, senescence, cell motility, differentiation, and proliferation [107,109].

Kinase imbalance may be a secondary cause of degeneration in adult-onset SMA, as BICD2 and DYNC1H1 mutations, which have been increasingly associated with abnormal kinase signaling that worsens motor neuron dysfunction beyond impaired axonal transport [110]. Mutations in BICD2 and DYNC1H1 disrupt the transport of RNP granules and signaling endosomes, thereby indirectly triggering stress-responsive kinases like p38 MAPK, ERK1/2, and AKT cause of the control cytoskeletal stability, synaptic maintenance, and neuronal survival [111]. In DYNC1H1 mutant neurons, dysregulated ERK signaling modifies neurotrophic signaling and growth cone dynamics, whereas BICD2 dysfunction causes GSK3β hyperactivation, which contributes to axonal degeneration and NMJ instability [112]. Long-term kinase activation impairs mitochondrial homeostasis and axon guidance, as confirmed by iPSC-derived motor neurons, which connect transferring abnormalities to more general signal transduction failure [113]. In particular phosphorylation networks may be targeted therapeutically.

### 2.4. DNA Damage and Repair Deficits

Genomic stability is crucial for neurons, which are vulnerable to cumulative DNA damage due to high metabolic demands and limited regenerative capacity. Recent studies reveal that impairments in DNA damage response (DDR) and repair pathways play a significant role in MNDs such as ALS, ALS-FTD, PLS, HSP, MSP, SMARD1 and SBMA [114,115]. These deficits stem not only from secondary cellular stress and mitochondrial dysfunction, but also from disruptions in RBPs and transport machinery interacting with chromatin and DNA repair complexes [116]. Mutant proteins including TDP-43, FUS, VCP, and HNRNPA1, typically linked to cytoplasmic aggregation, are now recognized for their roles at DNA damage foci, affecting the NHEJ, BER, and SSBR pathways [117]. Their mislocalization or loss-of-function leads to persistent DNA lesions, genomic instability, and apoptotic signaling in neurons. Impaired DDR further amplifies neuroinflammation, mitochondrial dysfunction, and protein misfolding, driving degeneration [118]. These insights position DNA repair deficits at the convergence of multiple pathogenic processes in MNDs and highlight their potential as biomarkers and therapeutic targets.

ALS is increasingly recognized as involving significant DNA damage and repair dysfunction, contributing to motor neuron vulnerability. DNA repair pathways, such as single-strand break repair, NHEJ, and HR, involve important proteins like TDP-43, FUS, and C9orf72. However, their mislocalization or aggregation blocks the recruitment of XRCC1, BRCA1, and RAD51 to damage sites, resulting in persistent DNA double-strand breaks and genomic instability [117]. C9orf72-derived DPRs disrupt ATM/ATR signaling and induce R-loop accumulation with exacerbating genomic compromise [119,120]. These deficits are particularly detrimental in post-mitotic motor neurons because of limited repair capacity. Postmortem tissues and iPSC models provide evidence of widespread DDR dysfunction associated with T cell-mediated neuroinflammation, oxidative stress, and mitochondrial impairment [121]. Ultimately genomic maintenance as a potential therapeutic target and place deficiencies in DNA repair could be a key factor in the pathophysiology of ALS.

Transcriptome and histopathological analyses of PLS show elevated markers like γH2AX and 53BP1 in degenerating corticospinal neurons, indicating genomic stress, even though they lack the characteristic cytoplasmic inclusions of ALS [20]. TDP-43 and dysregulated RBPs may hinder nuclear trafficking of repair factors and interfere with the communication between BER and transcriptional stress responses [122]. Oxidative stress linked to mitochondrial dysfunction likely exacerbates repair deficits, especially in long-projection pyramidal neurons [123]. Early treatment approaches with focus on genomic maintenance and point to a chronic maybe effective by subclinical DNA repair deficit in PLS to selective upper motor neuron vulnerability [20].

HSP has been linked to secondary DNA damage and repair deficits contributing to progressive axonal degeneration. DNA damage accumulation, increased γH2AX foci, and downregulation of ATM and BRCA1 signaling are signs of compromised DSB repair caused by SPG11 deficiency, which also affects chromatin remodeling, lysosomal function, and autophagy-dependent DNA clearance [23,124]. Through an increase in mitochondrial reactive oxygen species (ROS), a secondary base excision repair (BER) defect in corticospinal axons with an indirect impairment of DNA repair, SPG7 dysfunction intensifies oxidative stress [25]. The potential DNA repair enhancement and ROS mitigation could be used as therapeutic strategies, indicating that oxidative stress-exacerbated DNA repair deficits contribute to upper motor neuron vulnerability in complex HSP forms [125,126].

SMARD1 involves deficits in DNA damage repair that contribute to motor neuron degeneration. IGHMBP2 is necessary for genomic stability because it promotes transcription termination, DNA replication, and R-loop resolution, all protecting against replication stress and DNA Double-Strand Breaks (DSBs) [26]. IGHMBP2 loss affects the recruitment of DNA repair proteins like RAD51, BRCA1, and γH2AX, which impacts the BER and Homologous Recombination (HR) pathways [127,128]. Oxidative stress and mitochondrial malfunction exacerbate these defects, leading to an increase in DNA lesions in motor neurons. Transgenic mouse models and SMARD1 motor neurons derived from iPSCs validate apoptosis, cell cycle arrest, and persistent DNA damage foci, suggesting a disruption in nuclear and cytoplasmic genome maintenance [129]. A novel therapeutic approach to slow neurodegeneration in SMARD1 may be to improve DNA repair.

MSP is increasingly recognized for involving not only protein aggregation and RNA dysregulation but also DNA damage and repair deficits. By removing failed repair proteins and attracting 53BP1 and BRCA1 to damage sites, VCP promotes chromatin remodeling and DSB repair [130,131]. These processes are compromised by mutations, which result in genomic instability and long-lasting DNA damage in muscle and motor neurons. Although they are mainly RNA-binding proteins, hnRNPA1 and hnRNPA2B1 also interact with DDR regulators to support BER and R-loop resolution [132]. These proteins’ mutations increase neuronal vulnerability by causing mislocalization, poor recruitment to damage foci, and dysregulated DDR transcripts [133]. Targeting DDR pathways may be a useful addition to aggregation-based therapies, as iPSC-derived MSP models verify that degeneration is caused by disruptions in RNA metabolism, DNA repair, and protein clearance [134].

SBMA produces polyQ-expanded AR that not only forms toxic aggregates but also disrupts DDR mechanisms. Particularly in situations of oxidative or androgen-induced stress, mutant AR impairs the recruitment of crucial repair proteins, such as ATM, ATR, and BRCA1, to DNA damage sites [135]. Due to transcription-replication conflicts, R-loop accumulation, and compromised DSB repair, this leads to genomic instability and heightened neuronal susceptibility [135]. Furthermore, mutant AR increases motor neurons’ sensitivity to DNA damage by interfering with co-regulators of BER and homologous recombination, including p53-binding protein 1 (53BP1) and RAD51 Recombinase [136]. deficiencies in DNA repair as a fundamental aspect of SBMA pathogenesis and support DDR-targeted therapies for managing neuromuscular degeneration.

IBMPFD involves disrupted DDR and repair mechanisms, including proteostasis and autophagy defects. VCP keeps the genome stable by promoting chromatin remodeling, DDR factor recruitment, and the elimination of stopped repair proteins [130]. Through disrupted recruitment of 53BP1, BRCA1, and RNF8 to damaged chromatin, pathogenic mutations impair DSB resolution, preventing HR and Non-Homologous End Joining (NHEJ) [131,137]; this leads to persistent DNA lesions, genomic instability, and apoptotic signaling, especially in muscle and neural tissues. In IBMPFD models, VCP-related DDR defects increase degeneration by interacting with mitochondrial oxidative stress and protein aggregation [138]. In IBMPFD, DNA repair dysfunction is a major pathogenic mechanism and a possible therapeutic target by highlighting VCP as a central regulator of nuclear and cytoplasmic homeostasis [139].

Adult-onset SMA research indicates that beyond disrupted axonal transport, mutations contribute to secondary DNA damage and impaired repair in motor neurons. Both BICD2 and DYNC1H1 are essential for trafficking components of the DDR machinery, including DNA repair proteins and chromatin remodelers [140]. Defective DSB repair and genomic instability result from DYNC1H1 mutations that affect retrograde transport, limit the recruitment of 53BP1 and γH2AX to DNA damage sites, and decrease the nuclear import of DNA repair proteins [84]. Similarly, BICD2 dysfunction disrupts cell cycle regulation and ATR–CHK1 signaling, which are crucial for neuronal survival under replication stress [141,142]. These deficiencies promote motor neuron degeneration and worsen oxidative stress, while improving aSMA’s ability to repair DNA could be a useful treatment approach for reducing neurodegeneration brought on by ongoing genotoxic stress [142].

### 2.5. Mitochondrial Dynamics and Dysfunctions

Mitochondria are central to neuronal function, providing energy, buffering calcium, regulating apoptosis, and managing oxidative stress. Motor neurons have long axons and high metabolic demands, and maintaining homeostasis requires precise regulation of mitochondrial dynamics, including fission, fusion, transport, and mitophagy. As a primary cause of degeneration in MNDs such as ALS, PLS, HSP, SBMA, and MSP, mitochondrial dysfunction is becoming more widely stated. This is frequently connected to genetic mutations that impact organelle structure, turnover, and bioenergetics [143,144]. Axonal and synaptic functions are impaired by changes in mitochondrial morphology and distribution, whereas neuroinflammation is triggered by increased ROS production due to compromised mitophagy and OXPHOS [145]. Via protease dysfunction, ER–mitochondria uncoupling, transport defects, and fission/fusion abnormalities, mutations in indirect modulators (e.g., VCP, IGHMBP2, AR) and mitochondrial regulators (e.g., SPG7, CHCHD10, SOD1, DYNC1H1), all impair quality control [146]. Neurotoxicity is further amplified in patient-derived neurons and models by interactions between mitochondrial stress and RBPs or kinase signaling. Particularly, mitochondrial dysfunction is a prevalent pathogenic characteristic of various MNDs, emphasizing bioenergetic modulation, mitochondrial biogenesis, and autophagic clearance as potential therapeutic targets [147].

ALS is caused by a complex mitochondrial dysfunction that degenerates both upper and lower motor neurons. SOD1, TDP-43, FUS, and C9orf72 interfere with quality control, bioenergetics, and mitochondrial dynamics. TDP-43 and FUS aggregates interfere with mitochondrial RNA processing and import, while mutant SOD1 mislocalizes to mitochondria, affecting the electron transport chain and raising ROS [144]. DPRs generated from C9orf72 contribute to neurotoxicity by causing mitochondrial fragmentation, membrane potential loss, and compromised calcium buffering [148]. Synaptic energy deficiencies result from disrupted axonal trafficking of mitochondria triggered by changes expression of kinesin, dynein, and Miro [149]. Mutant VCP disrupts mitophagy, leading to an accumulation of malfunctioning mitochondria and stress on neurons [148,149]. Accordingly, mitochondrial dysfunction is a major early factor in the pathophysiology of ALS; meanwhile, mitophagy modulation, mitochondrial biogenesis enhancement, with specific antioxidants, are potential therapeutic approaches.

The pathophysiology of PLS is attributed to mitochondrial dysfunction. Transcriptome and proteome analyses of motor cortex tissue reveal changes in genes related to OXPHOS, fission/fusion, and calcium handling, further indicating metabolic stress in cortico-spinal neurons, despite the lack of direct genetic mitochondrial associations [150]. In preclinical models and patient samples, decreased complex I and IV activity and elevated oxidative stress markers, such as 4-HNE and nitrotyrosine, point to compromised mitochondrial respiration [151]. iPSC-derived upper motor neurons exhibit impaired calcium buffering, reduced membrane potential, and early mitochondrial fragmentation. The need for mitochondria-targeted therapies is highlighted by these alterations, which are less pronounced than in ALS but indicate that chronic mitochondrial dysfunction plays a role in axonal degeneration and synaptic failure.

SPG7, which encodes the mitochondrial protease paraplegin, is one of the HSP subtypes that most prominently involves mitochondrial dysfunction. Its mutation impairs mitochondrial dynamics, raises oxidative stress, and disturbs the respiratory chain, particularly in long corticospinal axons. Axonal degeneration and synaptic failure in SPG7-deficient neurons are caused by aberrant mitochondrial morphology and decreased ATP production [152]. On the other hand, by interfering with the lysosome–mitochondria axis, SPG11 disrupts lysosomal trafficking and autophagy, which indirectly impairs mitochondrial function by causing defective mitophagy, the accumulation of depolarized mitochondria, and increased ROS production [153]. SPG11 mitochondrial dysfunction is further supported by abnormal mitochondrial morphology and deficiencies in calcium buffering. All these findings point to primary (SPG7) or secondary (SPG11) mitochondrial dysfunction as a major cause of axonal vulnerability in HSP, indicating that treatments focusing on mitochondrial stress may be advantageous for different subtypes.

While helicase dysfunction and RNA processing are characteristics of SMARD1, mitochondrial impairment is a major factor in its pathophysiology. In particular, in high-demand motor neurons, IGHMBP2 deficiency impairs OXPHOS, depletes ATP, increases ROS, and interferes with mitochondrial protein translation and ribosomal integration [154]. Transgenic models and neurons derived from SMARD1 iPSC exhibit defects in mitophagy, loss of membrane potential, and fragmented mitochondria [129]. Neuronal stress is exacerbated by impaired mitochondria–ER interactions that further interfere with calcium signaling [155]. Therapeutic strategies that emphasize mitochondrial dysfunction as a major consequence of helicase loss, with a focus on mitochondrial bioenergetics and proteostasis.

MSP pathogenesis is associated with mitochondrial dysfunction, specifically through impaired mitophagy, oxidative stress, and disrupted organelle trafficking. Mutant VCP compromises mitochondrial quality control by interfering with PINK1/Parkin-mediated clearance of damaged mitochondria, which leads to the accumulation of organelles that produce reactive oxygen species [46,156]. Reduced ATP production, mitochondrial membrane potential, and fragmentation are observed in MSP fibroblasts and models, particularly in motor neurons and muscle cells [156]. Mutations in hnRNPA1 and hnRNPA2B1 affect RNA metabolism, which is necessary for mitochondrial protein synthesis, further impairing mitochondrial function. In addition to reducing mitochondrial ATP production and impairing Ca^2+^ exchange, disruption of ER-mitochondria contacts increases cellular stress and excitotoxic susceptibility [157]. The potential of mitochondrial stabilization and mitophagy enhancement as therapeutic targets in MSP is highlighted by the close relationship between these mitochondrial deficits, protein aggregation, and autophagy failure.

One of the main causes of motor neuron and muscle degeneration in SBMA is mitochondrial dysfunction brought on by a polyQ expansion in the AR gene. The integrity, bioenergetics, and oxidative balance of the mitochondria are disrupted by PolyQ-expanded AR, which also affects the electron transport chain (ETC), lowers ATP synthesis, increases ROS, and depolarizes the membrane [158]. Additionally, AR aggregates disrupt calcium homeostasis, which causes mitochondrial apoptosis by interfering with ER-mitochondrial communication and mitochondrial protein import. These aggregates affect autophagy, the ubiquitin-proteasome system, and impaired mitophagy triggers an accumulation of malfunctioning organelles, which further impairs muscle and neuronal function [159]. While androgens make mitochondrial stress worse, androgen-deprivation treatments can slow the progression of disease and partially restore mitochondrial dynamics [160]. Since mitochondrial dysfunction is a key mechanism in SBMA, hormonal modulation and mitochondria-targeted therapies may be effective treatments.

IBMPFD shows significant mitochondrial dysfunction contributing to motor neuron and muscle degeneration. Energy failure and ROS accumulation result from a mutation in VCP, which controls mitophagy and hinders the removal of damaged mitochondria [46]. In muscle and spinal motor neurons, patient tissues and VCP-mutant models exhibit changed mitochondrial morphology, fragmentation, and decreased membrane potential [161]. Decreased VCP function also interferes with mitochondria–ER interactions, which leads to calcium imbalance, activation of the Unfolded Protein Response (UPR), and increased vulnerability to cell death. Muscle atrophy and neurodegeneration are made worse by these abnormalities in conjunction with impaired ATP synthesis, oxidative stress, and inflammatory signaling [156]. VCP plays a crucial role in maintaining organellar homeostasis, and the combination of mitochondrial dysfunction, proteostasis collapse, and autophagy defects makes mitochondrial stabilization a viable therapeutic target for IBMPFD.

Adult-onset SMA is also associated with axonal transport alterations and significant mitochondrial dysfunction. Key elements of the dynein–dynactin motor complex are necessary for retrograde mitochondrial trafficking, which is encoded by the BICD2 and DYNC1H1 genes. Their malfunction leads to mitochondrial mislocalization, particularly in NMJs and distal axons, in synaptic vulnerability and localized energy failure [162,163]. Whereas BICD2 mutations affect mitochondrial transport and anchoring by interfering with microtubule interactions, while mutant DYNC1H1 causes mitochondrial swelling, fragmentation, and membrane potential loss in spinal motor neurons [164]. Reduced ATP availability, increased ROS production, and the activation of stress pathways like UPR and mitochondrial apoptosis, which are all consequences of energy deficits resulting from impaired mitochondrial distribution [165]. Targeting mitochondrial trafficking, bioenergetics, and quality control may be therapeutically beneficial in adult-onset SMA, as a direct consequence of axonal transport failure.

### 2.6. Immune Activation, T Cell Involvement, and Glial Contribution

Although MND progression has historically been ascribed to intrinsic neuronal vulnerability, new research shows that immune dysregulation, including T cell infiltration and glial dysfunction, plays an active role [166] Early distal axonal vulnerability and dysregulated glial cell–motor neuron interactions are increasingly recognized [167] Transcriptomics, single-cell RNA sequencing, and imaging reveal astrocytes, microglia, and infiltrating T lymphocytes as important participants in the neuroimmune landscape that impact motor neuron survival and plasticity in familial and sporadic MNDs like ALS, PLS, SBMA, and MSP. While activated microglia and astrocytes lose defensive capabilities like glutamate clearance and trophic support, they take on pro-inflammatory profiles by secreting TNF-α, IL-1β, and complement proteins [10]. Notably, in ALS, CD4^+^ and CD8^+^ T cells either worsen neuroinflammation or, occasionally, control it via regulatory T cells (Tregs) [168]. Via cGAS-STING, RIG-I, and interferon responses, mutations in VCP, HNRNPA1, SPG11, and C9orf72 also activate innate immune pathways [169]. Immune-targeted therapies, like altering T cell subsets, microglial phenotypes, or cytokine signaling, have the potential to be effective treatment options because immune activation is not just a byproduct but an active, disease-modifying process in MNDs.

As a neuroimmune disease, ALS is well known to be influenced by immune activation, T cell infiltration, and glial dysfunction. Through increased expression of NLRP3 inflammasome components, TNF-α, and IL-1β, microglia in ALS change from an initially protective to a chronically activated, neurotoxic phenotype that contributes to synaptic pruning and oxidative stress [10,170,171]. Additionally, ALS causes astrocytes to lose their homeostatic functions, lowering glutamate uptake (because of decreased EAAT2/GLT1) and releasing pro-inflammatory mediators that contribute to the death of motor neurons [172]. Importantly, regulatory T cells (Tregs) and CD4 and CD8 T cells infiltrate the motor cortex and spinal cord, where their balance affects the progression of the disease. While cytotoxic T cells are linked to a faster decline, Tregs are linked to a slower progression [168]. It has been demonstrated that ALS-related genetic mutations like C9orf72, SOD1, and TARDBP activate innate immune pathways like cGAS-STING, boosting interferon responses and inflammasome activity [169]. Immune dysfunction, particularly glial and T cell imbalance, is a primary, modifiable factor in ALS, enhancing the case for immunomodulatory treatments that are presently being studied in clinical settings.

PLS involves mild but substantial neuroimmune activation, especially in the motor cortex. Even in the absence of extensive neuronal loss, a reactive glial state is indicated by microglial activation and increased pro-inflammatory cytokines (e.g., IL-6, TNF-α) [173,174]. Astrocytes exhibit glutamate dysregulation and stress, which may increase the vulnerability of corticospinal neurons [175]. Although less noticeable than in ALS, T cell infiltration, particularly of CD8^+^ cells, has been found in cortical tissue and may exacerbate dysfunction in upper motor neurons [168]. PLS transcriptomic profiling demonstrates immune involvement through altered MHC class I expression and upregulation of immune-related genes [171]. Glial and T cell-mediated immune responses contribute to the pathophysiology of PLS, indicating that immune-modulating therapies may have therapeutic potential even in this slower-progressing motor neuron disease setting.

Emerging evidence in HSP, especially in complex forms like SPG11 and SPG7, emphasizes the role of glial dysfunction and immune activation as contributors to corticospinal tract degeneration. Chronic inflammation has been suggested by the detection of microglial activation, elevated IL-1β, TNF-α, and complement C3 in postmortem tissue and SPG11 models [176]. Lack of SPG11 is associated with lysosomal dysfunction, which results in an accumulation of unprocessed autophagic material, triggering innate immunity and advancing axonal degeneration [98]. In SPG7, glial excitotoxicity is increased by mitochondrial stress, which also causes oxidative damage and astrocyte reactivity [99]. Transcriptome studies show upregulation of MHC I/II in complex HSP motor cortex, suggesting possible adaptive immune engagement, even though T cell infiltration is less obvious than in ALS [177]. Although immune and glial factors influence the progression of HSP disease, they could be targets for treatment.

According to recent research in SMARD1, glial responses and immune activation are secondary factors in the disease development. IGHMBP2 deficiency causes misprocessed RNA and stalled ribonucleoprotein complexes to accumulate, which in turn triggers innate immune responses via type I interferon signaling in motor neurons and RIG-I-like receptor routes [26,178]. In mouse models, glial reactivity appears before overt neurodegeneration, as evidenced by increased levels of TNF-α and IL-6, as well as early microglial priming and astrogliosis [129]. Markers have been found in peripheral nerves and spinal tissue, suggesting peripheral immune involvement, even though T cell infiltration is less obvious than in ALS [28]. In the larger context of neuroimmune-modulated motor neuron disorders, SMARD1 may increase motor neuron stress and degeneration through immune dysregulation, even as a secondary consequence [169].

Glial dysfunction and immune system activation are key components of MSP’s multisystem neurodegenerative pathology with mutations in VCP, HNRNPA1, and HNRNPA2B1. By disrupting autophagy and proteostasis, mutant VCP increases neuroinflammation by accumulating misfolded proteins and activating the NLRP3 inflammasome, TLR3, and cGAS-STING pathways [31,169]. Especially in motor areas, reactive microglia and astrocytes increase oxidative stress markers and pro-inflammatory cytokines (IL-1β, IL-6, and TNF-α) [10]. MSP patient tissues and models have shown T cell infiltration, including CD8^+^ and CD4^+^ cells, indicating adaptive immune involvement [179]. Furthermore, mislocalized hnRNPA1/A2B1 and changed stress granule dynamics enhance chronic inflammation by contributing to ISG expression [31]. Glial and immune mechanisms as active modulators in MSP, providing opportunities for immunomodulatory treatments to supplement approaches with protein aggregation targets.

Beyond SBMA motor neuron and muscle pathology, immune activation and glial involvement are increasingly recognized. Meanwhile, in both patient tissue and AR-expanded mouse models, there is increased IL-6, TNF-α, and complement proteins, as well as astrocyte and microglial activation, particularly in the brainstem and spinal cord [106,180]. The NF-κB pathway is activated, and inflammation increases when polyglutamine-expanded AR assembles into nuclear and cytoplasmic aggregates that interact with innate immune sensors [181]. Even though T cell infiltration is not as noticeable as in ALS, immune profiling indicates peripheral immune dysregulation in skeletal muscle due to elevated MHC class I expression and changed cytokine signaling [182]. Moreover, androgens alter T cell function and microglial phenotype in SBMA, which may account for sex-specific variations in the disease progression because males are more likely to experience immune suppression [183]. Immunomodulatory and anti-inflammatory treatments and androgen-targeted strategies further establish the role of the neuroimmune direction in SBMA.

Concerning IBMPFD, neurodegeneration and multisystem disorders are largely caused by immune activation and glial dysfunction. Through NF-κB, NLRP3 inflammasome, and cGAS-STING pathways, mutant VCP triggers innate immune responses in neural and glial cells by interfering with autophagy, proteostasis, and mitochondrial function [31,169]. Studies on animals and postmortems show microgliosis and astrogliosis, along with increased levels of IL-1β, IL-6, and complement C3, contribute to the motor neuron degeneration and muscle fibers [179]. IBMPFD muscle and CNS tissue contain D8^+^ and CD4^+^ T cells, indicating adaptive immune engagement triggered by accumulated protein and nucleic acid debris [183]. The role of T cell and glial dysfunction as major contributors to neuroinflammation, strengthening the case for immune-modulatory and autophagy-restoring treatments in IBMPFD [184].

In adult-onset SMA, immune and glial activation play a secondary role in the development of the disease. Defects in retrograde transport, as well as impaired retrograde trafficking and mislocalized RNA-protein complexes, also cause chronic neuronal stress, which activates astrocytes and microglia in spinal motor circuits [185,186,187]. IL-1β, CCL2, TNF-α, microglial priming, and astrogliosis are elevated in dyneinopathy models before overt axonal degeneration [188]. Immunological profiling shows increased interferon-stimulated genes and MHC class I molecules in neurons and glia, suggesting a pro-inflammatory state, even though T cell infiltration is restricted in aSMA [189]. Furthermore, immune modulation could be a possible supplementary treatment to axonal transport restoration, with a focus on glial reactivity and stress signaling [185].

## 3. Pharmacological Treatments and Clinical Trials

Mechanism-based therapeutic development has replaced symptomatic care as a result of our increasing understanding of the convergent molecular mechanisms in MNDs. Although the variety of genetic mutations poses difficulties, pathway-targeted therapies have become possible due to common downstream pathologies like kinase signaling abnormalities, mitochondrial dysfunction, RNA dysregulation, proteostasis failure, and neuroinflammation. Although the MNDs discussed ALS, PLS, HSP, SBMA, SMARD1, MSP, IBMPFD, and aSMA are rare and varied, they all have neuroaxonal degeneration in common, which makes mechanism-targeted therapies possible. Treatment has historically centered on gene-specific therapies or symptomatic alleviation, but the new discoveries about the mechanisms underlying shared diseases have directed attention towards tactics that target shared molecular pathways (Table 4).

### 3.1. RNA-Targeted Therapies and Antisense Oligonucleotides (ASOs)

RNA-targeted therapies, particularly ASOs, that alter splicing, inhibit harmful RNA species, or restore transcript balance, are among the most revolutionary methods in MND therapeutics. ASOs’ potential in motor neuron disorders has been confirmed by Nusinersen, which increases SMN protein by altering SMN2 splicing, while it has also shown significant efficacy in treating spinal muscular atrophy [190]. Similar to this, Tofersen inhibits the translation of mutant SOD1 mRNA, which may slow the ALS progression [191]. Building on these successes, studies are developing ASO pipelines for genes linked to uncommon MNDs, such as HNRNPA1/A2B1 (stress granule-associated MSP), FUS (mislocalized RNA-binding protein), SPG11 (RNA granule turnover), IGHMBP2 (helicase-linked SMARD1), and C9orf72 (repeat-expanded RNA foci and DPRs) [192]. One of the main obstacles to RNA-based therapy is being addressed by improvements in CNS bioavailability and neuron-specific uptake brought about by advancements in delivery systems, such as intrathecal nanoparticles, viral vectors, and peptide-conjugated oligos [193].

### 3.2. Proteostasis Restoration Strategies

Since protein misfolding and aggregation play a major part in conditions like ALS, SBMA, and MSP, re-establishing proteostasis is an important therapeutic objective. Strategies include disaggregase tactics based on heat shock protein (HSP) chaperone modulation and autophagy enhancers (such as rapamycin and trehalose) that enhance the removal of misfolded proteins [194]. Modifying the UPR with VCP inhibitors or ER stress modulators has demonstrated preclinical effectiveness in lowering protein accumulation, re-establishing organelle dynamics, and enhancing motor function in animal models of VCP-linked disorders like MSP and IBMPFD [41].

### 3.3. Mitochondrial Therapeutics

Mitochondrial dysfunction is particularly noticeable in HSP (SPG7), SBMA, SMARD1, and MSP, leading to axonal degeneration through oxidative stress, impaired ATP production, and calcium-protection failure mechanisms. Therapeutic candidates that target oxidative phosphorylation and ROS scavenging include coenzyme Q10, MitoQ, and nicotinamide riboside [195]. Although Bezafibrate and Pioglitazone improve mitochondrial biogenesis and metabolic resilience, agents such as PINK1/Parkin activators promote mitophagy and mitochondrial recycling [196,197]. These strategies aim to improve axonal energy supply and reduce degeneration at early stages.

### 3.4. Kinase Signaling Modulators

Abnormalities in kinase signaling, such as activation of p38 MAPK, GSK3β, and JNK, are linked to cytoskeletal disruption, stress granule persistence, and hyperphosphorylation of RNA-binding proteins in ALS, PLS, and aSMA. A p38 MAPK inhibitor called Neflamapimod is being studied in frontotemporal dementia and ALS, and it exhibits promise in PLS and MSP models [198]. GSK3β inhibitors, initially investigated in Alzheimer’s disease, are becoming more popular for their ability to stop TDP-43 aggregation and restore microtubule dynamics [95]. These substances are a manageable class of small molecules that might work especially well for MND patients with hyperactivated kinase signatures.

### 3.5. Immune Modulation Strategies

Neuroinflammation and T cell–mediated toxicity, once considered secondary phenomena in MNDs, are now recognized as therapeutic targets. IL-6 inhibitors, low-dose IL-2 for regulatory T cell (Treg) expansion, and glial suppressants like Ibudilast and minocycline are being studied in ALS and SBMA trials [10]. Even in MNDs like PLS and SPG11-linked HSP, where classical inflammatory pathology is absent, there is increasing evidence that microglial and T cell dysregulation also play a role in the disease’s progression [199]. ASOs or proteostasis modulators may be used in conjunction with immune-targeted therapies to treat a broader range of MND diseases.

### 3.6. Integrating Biomarkers and Precision Trial Design

Strong outcome indicators and better patient stratification are essential for mechanism-based therapies to be effective. The use of biomarkers in early-stage trials, such as cytokine signatures, RNA-binding protein mislocalization profiles, and NfL, is growing. Meanwhile, adaptive platforms such as TRICALS and HEALEY-ALS are promising models for extending trial infrastructure to rare MND subgroups that are defined by pathophysiology and genetics [200]. This kind of integration is crucial to moving from general neuroprotection to precision therapies.

### 3.7. Emerging Clinical Trials Across the MND Spectrum

Recent translational research has initiated a new generation of clinical trials targeting both common and disease-specific mechanisms across MNDs. In ALS, the AMT-162 gene therapy trial (uniQure, Phase I/II) employs an AAVrh10-delivered microRNA to silence mutant SOD1, with the first patient dosed in October 2024 and subsequent dose escalation approved following favorable safety outcomes [201]. To address synaptic dysfunction, SPG302, administered as a small molecule, has entered Phase II testing with the goal of enhancing synaptic connectivity and neuroplasticity in ALS [202]. Furthermore, NUN-004, a biologic that targets ephrin signaling, is undergoing evaluation in a Phase I/Ib trial; preliminary findings show that it is effective in slowing the progression of ALS and has good safety, tolerability, and a favorable pharmacokinetic profile [203]. In SMA, five-year follow-up data on Onasemnogene Abeparvovec (Zolgensma), an AAV9-based gene therapy targeting SMN1, continues to demonstrate sustained survival and motor improvements with a single dose [204,205]. For SMARD1, preclinical studies on AAV9-mediated IGHMBP2 gene replacement have shown promising results, with a first-in-human trial anticipated in late 2024 [205]. In SBMA (Kennedy’s Disease), the investigational compound AJ201 is currently in a Phase 1/2a trial assessing its safety, tolerability, and therapeutic impact on muscle and physical function. Preliminary data suggest that AJ201 may improve clinical outcomes in SBMA patients [206,207]. More geographic and phenotypic representation for MSP related to VCP has been made possible by the inclusion of a remote-only cohort. Results from future trials will provide information on the clinical outcome assessments’ (COAs’) responsiveness, feasibility, and sensitivity to change over a two-year period [208]. In HSP particularly SPG11 and SPG15, compounds such as Naringenin have been identified as enhancers of lysosomal reformation downstream of mTOR signaling [209]. This strategy has shown potential in correcting lysosomal dysfunction in preclinical models and supports the development of Phase II trials targeting these subtypes [209].

Gene-by-gene approaches to treating MNDs are giving way to shared-mechanism strategies that focus on immune modulation, kinase signaling, mitochondrial health, proteostasis failure, and RNA dysregulation. The best chance for disease modification in the near future is provided by the combination of multitargeted pharmacology and biomarker-driven clinical trials, even though no existing treatment fully addresses all aspects of neuroaxonal degeneration. MND disease sample groups and larger neurodegeneration research projects must continue to collaborate to turn these tactics into successful treatments.

However, several critical limitations continue to hinder the successful development and translation of therapies for MNDs. The lack of reliable and disease-specific biomarkers, particularly for early diagnosis and treatment response, remains a major challenge. Additionally, the predictive value of current animal and cellular models is limited because they frequently fall short of accurately replicating the phenotypes of human diseases. Small patient populations, disease variability, and the challenge of tracking progress over brief periods are additional limitations on clinical trial design.

## 4. Discussion

One of the most compelling insights to emerge from recent mechanistic studies is that RNA dysregulation plays a direct, causative role in axonal degeneration across motor neuron diseases. Disruption of RNA-binding proteins such as TDP-43, FUS, and IGHMBP2 affects nuclear RNA processing and compromises the transport and local translation of key transcripts within axons. This leads to ribosome depletion, loss of axonal proteins essential for cytoskeletal stability and mitochondrial support, and ultimately, structural axon failure. For example, TDP-43 pathology in ALS has been shown to reduce axonal levels of STMN2 and NEFL mRNAs, impairing axon outgrowth and maintenance [18,19,48]. At the same time, SMARD1 models with IGHMBP2 deficiency exhibit stalled translation and early neuromuscular junction disassembly even before motor neuron death occurs [51,52]. These findings, supported by studies in patient-derived motor neurons and in vivo models, strengthen the rationale for RNA-targeted therapies aimed at preserving axonal integrity and slowing disease progression in MNDs [6,49,53].

MNDs have traditionally been studied as distinct clinical and genetic disorders, limiting understanding of their shared mechanisms and slowing progress toward effective treatments. This review bridges this gap by synthesizing mechanistic insights across MND diseases such as ALS, PLS, HSP, SBMA, SMARD1, MSP, IBMPFD, and aSMA, revealing how converging molecular pathways underpin disease progression. Despite differences in genetic causes and clinical presentations, these disorders share disruptions in RNA regulation, proteostasis, mitochondrial integrity, DNA repair, cell signaling pathways, and immune responses [4,5,6,7,8,9,10]. These disruptions culminate in progressive neuroaxonal degeneration, which is a key determinant of motor dysfunction and disability [3,8].

A fundamental finding is the identification of RNA dysregulation and prion-like protein aggregation as early drivers of disease. These processes trigger downstream effects, including impaired mitophagy, defective DNA repair, and activation of stress-responsive kinase signaling, contributing to neuronal dysfunction [4,12,13]. Importantly, mitochondrial dysfunction emerges not just as a secondary consequence but as a primary driver of neurodegeneration in some subtypes (e.g., SPG7, VCP-related MSP), emphasizing the need to target mitochondrial quality control in therapeutic strategies [9,25,144]. Moreover, immune activation, including T cell infiltration and glial responses, is increasingly recognized as a key factor, even in MNDs without inflammation, such as PLS, and SMARD1 [10,22,24,26].

From a pharmacological perspective, antisense oligonucleotides are already advancing in ALS and SMA, which show targeting toxic RNA species [194]. Autophagy enhancers and proteostasis modulators, such as trehalose, rapamycin, and VCP inhibitors, are being explored for their ability to clear misfolded proteins and restore cellular homeostasis [41]. Kinase inhibitors (e.g., targeting p38 MAPK, GSK3β, and ERK pathways) offer a means to correct dysregulated phosphorylation events implicated in neurodegeneration [198]. Immune-modulatory approaches, including IL-6 inhibitors, Treg therapies, and glial suppressants, are prospective for dampening neuroinflammation across MNDs [10].

This synthesis also calls for a shift in clinical trial design. Trials should move beyond single-gene stratification to include patients grouped by mechanistic profiles, integrating biomarkers of RNA dysregulation, mitochondrial dysfunction, proteostasis collapse, kinase signaling, and immune activation. Digital biomarkers, neurofilament levels, and advanced imaging modalities will be essential for the dynamic assessment of therapeutic efficacy [11,13,210]. Precision medicine approaches, integrating multi-omics data and patient-derived models, such as iPSC-derived neurons and organoids, will enable the identification of patient-specific vulnerabilities and enhance therapeutic targeting.

While this review highlights converging molecular mechanisms across the MND spectrum, important areas of divergence and ongoing debate must also be recognized. For instance, the extent and nature of neuroinflammation vary significantly between ALS, SMA, and SBMA. ALS exhibits robust microglial activation and T cell infiltration, often contributing to non-cell autonomous motor neuron injury [11], whereas SMA shows a more cell-autonomous degeneration pattern with limited peripheral immune involvement [205]. Similarly, the role of TDP-43 pathology remains inconsistent across SMA and SBMA models, while TDP-43 mislocalization is nearly universal in ALS [1,2], it is not a prominent hallmark in classical SMA, and in SBMA, mutant polyQ-expanded AR forms nuclear and cytoplasmic inclusions with distinct pathogenic cascades [4,206]. These discrepancies underscore unresolved questions regarding the contribution of glial cells, immune modulation, and selective vulnerability in different MND subtypes [5,6,207].

In summary, this integrated mechanistic view highlights the potential mechanisms of MNDs as platforms for advancing both scientific understanding and clinical interventions. By targeting shared molecular vulnerabilities with multi-pathway, precision-based therapies and optimizing clinical trial designs, we can accelerate the development of effective treatments for MNDs.

## 5. Future Directions

The disease mechanisms into effective treatments are critical for the future motor neuron disease research. Novel strategies with target several processes, including RNA-based treatments, medications that aid in the removal of damaged proteins and mitochondria, therapies that modify cell signaling, and medications that regulate the immune system, may be able to stop or slow the progression of disease. Advanced cell analyses, mini-organs, and nerve cells derived from stem cells are examples of laboratory models that will be used to pinpoint each patient’s unique vulnerabilities and find more accurate disease tracking indicators. Newer, more sensitive methods, such as digital tools and sophisticated scans, should be used in clinical trials, and patients should be grouped according to the molecular mechanism of their disease. Finally, research on MNDs not only benefits those who suffer from them but also sheds light on the etiology and potential treatments of other neurodegenerative diseases and specific mitochondrial disorders.

## 6. Conclusions

Motor neuron diseases, despite differences in genetic causes and clinical symptoms, share common underlying mechanisms that lead to progressive nerve cell damage. These include disruptions in RNA regulation, protein folding, mitochondrial function, DNA repair, cell signaling, axonal transport, and immune system activity. Together, these factors cause nerve cell degeneration and loss of motor function. Recognizing these shared processes highlights opportunities to target them with treatments that could help across different types of MNDs.

## Figures and Tables

**Table 1:** Major molecular mechanisms of axonal degeneration in MNDs.

Mechanism	Diseases	Molecular Features
RNA Dysregulation	ALS-FTD, SMARD1	TDP-43, IGHMBP2, EXOSC3
Protein Misfolding	SBMA, MSP, ALS	AR-polyQ, VCP, TDP-43
Mitochondrial Dysfunction	HSP, SMA, ALS	SPG7, DYNC1H1, SOD1
Kinase Signaling Defects	ALS, SBMA	p38, AKT, ERK dysregulation
Axonal Transport Failure	SMA, ALS-FTD	DYNC1H1, BICD2, IGHMBP2

Abbreviations: ALS, Amyotrophic Lateral Sclerosis; SBMA, Spinal and Bulbar Muscular Atrophy (Kennedy’s disease); MSP, Multisystem Proteinopathy; HSP, Hereditary Spastic Paraplegia; SMA, Spinal Muscular Atrophy; TDP-43, TAR DNA-binding Protein 43; IGHMBP2, Immunoglobulin Mu Binding Protein 2; EXOSC3, Exosome Component 3; AR-polyQ, Androgen Receptor with Polyglutamine Expansion; VCP, Valosin-Containing Protein; SOD1, Superoxide Dismutase 1; SPG7, Spastic Paraplegia Gene 7 (paraplegin); DYNC1H1, Dynein Cytoplasmic 1 Heavy Chain 1; p38, AKT, ERK, kinase signaling pathways (p38 mitogen-activated protein kinase, protein kinase B/AKT, extracellular signal-regulated kinase).

**Table 2:** Disease-specific molecular pathways underlying axonal degeneration in MNDs.

Disease	RNA Dysregulation	Protein Misfolding/Aggregation	Axonal Transport Defects	Mitochondrial Dysfunction	Glial/T Cell Immune Involvement
ALS-FTD spectrum	TDP-43 mislocalization, FUS mutations, C9orf72 RNA foci	TDP-43, SOD1, FUS aggregates	KIF5A, TUBA4A, C9orf72 affect transport	SOD1, C9orf72, and CHCHD10 impair ETC and calcium buffering	Astrocyte dysfunction, T cell infiltration, microgliosis
PLS	Shared RBP changes with ALS (e.g., TDP-43 pathology)	Less prominent, but misfolded neurofilaments may occur	Degeneration of long CST axons, slow axoplasmic flow	Emerging data suggest mild mitochondrial stress	Cortical microglia activation: immune transcripts upregulated
HSP (SPG11, SPG7)	SPG11 affects RNA metabolism, with potential spliceosome involvement	SPG11 may secondarily cause proteostasis stress	SPG11 affects autophagosomes and cargo delivery	SPG7 encodes mitochondrial protease; SPG11 impacts lysosome–mitochondria axis	Spastic paraplegia with variable neuroinflammation
SMARD1	IGHMBP2 mutation disrupts RNA helicase activity, and mRNA decay	IGHMBP2 loss leads to stalled ribosome-associated protein aggregates	Disrupted ribosome transport and NMJ targeting	Energy failure from ribosomal/mitochondrial collapse	Early microglial priming in the spinal cord: interferon signatures
MSP (VCP, HNRNPA1/2B1)	Mutant HNRNPA1/2B1 disrupts RNP granules, RNA export	VCP and RBPs form cytoplasmic inclusions	VCP mutations impair dynein-dependent cargo transport	Impaired mitophagy and ATP supply in VCP mutants	CNS inflammation, T cell-mediated degeneration
SBMA (Kennedy’s Disease)	AR polyQ expansion interferes with splicing and RBP dynamics	Misfolded AR protein aggregates in nuclei and cytoplasm	Impaired AR nuclear shuttling affects retrograde signaling	AR aggregates impair mitochondrial membrane integrity	Androgen-linked immunomodulation; possible glial stress
IBMPFD (VCP)	VCP impacts RNA surveillance and stress granule resolution	VCP-associated aggregates disrupt proteostasis	Autophagy and organelle trafficking are disrupted	Mitochondrial clustering and UPR activation in muscle and neurons	Neuroinflammation and glial reactivity in cortex and muscle

Abbreviations: HNRNPA1, Heterogeneous Nuclear Ribonucleoprotein A1; TDP-43, TAR DNA-binding Protein 43; FTD, Frontotemporal Dementia; FUS, Fused in Sarcoma; C9orf72, Chromosome 9 Open Reading Frame 72; AR, Androgen Receptor; CHCHD10, Coiled-Coil-Helix-Coiled-Coil-Helix Domain-Containing Protein 10; KIF5A, Kinesin Family Member 5A; TUBA4A, Tubulin Alpha-4A Chain; CST, Corticospinal Tract; MSP, Multisystem Proteinopathy; NMJ, Neuromuscular Junction; SMARD1, Spinal Muscular Atrophy With Respiratory Distress Type 1; ETC, Electron Transport Chain; UPR, Unfolded Protein Response; RNP, Ribonucleoprotein Complex.

**Table 3:** Prion-like aggregation in MNDs.

Diseases	Prion-Like Proteins/Aggregates	Mechanistic Implications
ALS	TDP-43, SOD1, FUS, C9orf72-associated DPRs	Template-directed misfolding, propagation via axons, and extracellular vesicles
PLS	TDP-43 (shared pathology with ALS), potential RBP granules	May amplify UMN pathology via chronic stress granule persistence
HSP (SPG11, SPG7)	SPG11-linked spatacsin may impair granule clearance, promoting indirect RBP aggregation	Likely contributes to proteostasis collapse in advanced HSP forms
SMARD1	Ribosome-associated stalling may promote noncanonical RNP aggregation	Stalled RNA–protein complexes may trigger aggregation
MSP (VCP, HNRNPA1/2B1)	Cytoplasmic stress granules with prion-like domains (hnRNPA1, hnRNPA2B1, VCP)	Self-propagating RBPs induce degeneration across motor neuron pools
SBMA (Kennedy’s Disease)	PolyQ-expanded AR forms nuclear/cytoplasmic inclusions with seeding capacity	Nuclear AR inclusions recruit splicing factors and disrupt proteostasis
IBMPFD (VCP)	VCP-linked aggregates exhibit prion-like spreading behavior in muscle and brain	Disruption of UPS and autophagy enables aggregate accumulation and spread
Adult-onset SMA (BICD2, DYNC1H1)	Not classical prion-like; possible cytoskeletal or transport-linked misfolding stress	Limited evidence suggests that aggregation may occur secondary to axonal transport stress

Abbreviations: DPRs, dipeptide repeat proteins; UPS, ubiquitin–proteasome system; RNP, ribonucleoprotein; RBP, RNA-binding protein; UMN, upper motor neuron; PolyQ-expanded AR, polyglutamine-expanded androgen receptor.

**Table 4:** Comprehensive pharmacological and clinical overview of MNDs.

Disease	Therapeutic Strategies	Clinical Trial Status	NfL as Biomarker	Disease Detection Method
ALS	Riluzole, Edaravone, Tofersen, AMX0035	Multiple Phase III trials, NfL endpoint	Validated in serum/CSF; prognostic marker	Clinical exam, EMG, MRI, genetic testing (SOD1, C9orf72)
PLS	Spasticity relief, Riluzole, neuroimaging biomarkers	Small observational studies, Riluzole trials	Elevated but lower than ALS; progression tracking	UMN signs on clinical exam, TMS, diffusion tensor imaging (DTI)
HSP (SPG11, SPG7)	Spasticity meds, mTOR/HDAC inhibitors, autophagy enhancers	Limited trials; EU registries (SPATAX)	Mildly elevated in complex cases	Spastic gait, family history, genetic panels, brain/spinal MRI
SMARD1	Gene therapy, ASOs, and ventilation support	Gene therapy in preclinical and early human use	Correlates with axonal loss; used in models	Neonatal hypotonia, phrenic nerve EMG, IGHMBP2 gene testing
MSP (VCP, HNRNPA1/2B1)	Proteostasis modulation, autophagy inducers	Emerging early-phase and biomarker trials.	Elevated in ALS-like forms; correlates with decline	Muscle biopsy, genetic testing (VCP, hnRNPA genes), family history
SBMA (Kennedy’s Disease)	Anti-androgens, ASOs, mitochondrial protectants	Phase III Leuprorelin completed; ASOs under study	Low/modest; progression marker in subtypes	Genetic confirmation of AR gene CAG repeat; EMG and hormonal profile
IBMPFD (VCP)	Autophagy and proteostasis targets, immunomodulators	Trial preparation underway	Elevated in cognitive/motor cases	Clinical triad (myopathy, Paget disease, FTD), VCP mutation testing
Adult-onset SMA (BICD2, DYNC1H1)	Transport modulators, ER-mitochondrial therapies	No clinical trials; iPSC modelling.	Potential marker, preclinical interest	Axonal neuropathy on EMG, genetic analysis (BICD2, DYNC1H1)

Abbreviations: AMX0035, a combination of sodium phenylbutyrate and taurursodiol for ALS treatment; SPATAX, European registry and research network for hereditary spastic paraplegias and related disorders; mTOR, mechanistic target of rapamycin; HDAC, histone deacetylase; NfL, neurofilament light chain; EMG, electromyography; MRI, magnetic resonance imaging; DTI, diffusion tensor imaging; TMS, transcranial magnetic stimulation; CSF, cerebrospinal fluid; iPSC, induced pluripotent stem cell; CAG, cytosine–adenine–guanine trinucleotide repeat.
